# Supplementary material for: Are carbon emissions trading and green financial instruments synergistic? -Comprehensive quantitative research based on content analysis
Source: PLoS One. 2024 Mar 7;19(3):e0298601. doi: 10.1371/journal.pone.0298601 (PMC10919601; doi:10.1371/journal.pone.0298601)
Supplement: S1 Data — (ZIP) [file pone.0298601.s001.zip › Data/地级市Z_F工作报告.zip!/┴╔─■/╜⌡╓▌╩╨/└┤╫╘╣1⁄2╓┌║┼í╛┬φ┐╦╩2╛▌═°í┐_2014.docx]

各位代表：

　　现在，我代表市政府向大会报告工作，请予审议，并请市政协各位委员提出意见和建议。

　　一、2013年工作回顾

　　2013年，面对严峻复杂的国内外经济形势，在省委、省政府和市委的正确领导下，我们坚定信心，苦干实干，较好地完成了市十五届人大一次会议确定的各项目标任务。

　　初步核算，全市地区生产总值实现1344.9亿元，增长8.8%；全社会固定资产投资完成952.1亿元，增长18.5%；规模以上工业增加值实现691.2亿元，增长9.8%；公共财政预算收入完成136.1亿元，增长3.5%；社会消费品零售总额实现494亿元，增长13.7%；实际利用外资11.4亿美元，增长13.5%；出口创汇21.2亿美元，增长21.1%；域外招商引资到位额857.6亿元，增长11.4%；城镇居民人均可支配收入25340元，增长10.2%；农民人均纯收入12137元，增长12.5%。

　　（一）沿海开发开放实现突破

　　世园会圆满成功。累计接待游客1119万人次，兑现了“世界给锦州一次机遇，锦州还世界一个精彩”的庄严承诺。国际园艺论坛、世界海洋城市论坛、世界海洋日暨全国海洋宣传日等重大活动亮点纷呈，全面提升了锦州的开放度、美誉度和影响力。广大建设者创造的“锦州世园精神”，已经成为推进全市发展的强大动力。

　　基础设施不断完善。锦州港中电投煤炭码头主体工程竣工，华润煤炭码头、中丝石化仓储项目进展顺利；实现货物吞吐量8500万吨、增长6.3%，集装箱运输90万标箱、增长12.5%。龙栖湾港区总体规划获批，前期工作进展顺利。锦州湾机场可研报告要件全部获批。滨海体育中心“两馆一校”投入使用，体育场主体工程竣工。工业园区、生活区道路管网等配套工程全面推进。渤海大学滨海校区、辽宁医学院、锦州师专、锦州中学新建迁建前期工作进展顺利。美化、绿化、亮化提档升级，开发区城中村搬迁改造步伐加快，滨海城市展现新的形象。

　　临港工业发展快速推进。辽宁嘉合精细化工一期60万吨碳四综合利用、海森堡石化135万吨燃料油、康泰10万吨润滑油等项目建成投产，中北石化60万吨润滑油、中石油天然气石油洞储等项目顺利推进，石化精细化工产业集群初具规模。龙栖湾差别化纤维一期25万吨聚酯、20万吨拉丝项目即将投产。格兰德分布式能源工业化应用项目落户大有。

　　服务业取得新进展。365客服基地一期工程主体完工，已开设服务席位3000个，微信114信息导航项目落户基地。新港湾酒店、东一雅阁国际酒店投入运营。十里金沙滩建成投入使用。天沐温泉度假村体验区对外运营，大有温泉生态城建设如期推进。

　　松山高新技术产业开发区、汤河子产业园区纳入省沿海经济带规划范围。百威英博一期30万吨啤酒、宝钛华神1万吨海绵钛、锦州钛业3万吨钛白粉扩能等项目建成投产。

　　（二）工业经济持续稳定增长

　　工业五项工程扎实推进。全年实施1000万元以上项目582个，其中亿元以上107个、10亿元以上8个。完成工业固定资产投资475.2亿元。规模以上工业企业总数达到839户。新增主营业务收入10亿元以上企业8户，总数达到39户。13个产业集群发展专项规划基本完成，省重点考核的5个产业集群实现销售收入1220亿元。完成并购项目4个。规模以上工业增加值能耗下降17%。

　　石化产业逆势上扬。锦州石化公司对全市的财税贡献好于往年。

　　光伏产业开始走出低谷。全行业累计实现产值145亿元。奥克阳光200MW多晶硅锭等5个项目建成投产。光伏公共服务研发平台、分布式光伏电站建设取得新进展，东旭太阳能、瑞德兴阳光伏项目成功签约。中美清洁能源合作项目示范市、国家可再生能源建筑应用示范市和国家新能源示范市创建工作全面推进。

　　工业运行质量继续向好。工业用电量稳定增长。召开11次地工产品产销衔接会，签购销协议52项。组织银企对接会活动，帮助企业融资45亿元。

　　科技创新上新台阶。高新技术产业增加值实现260.6亿元，增长33.9%。新引进海外研发团队项目11个。新认定国家级高新技术企业11家。新建省级重点实验室1家、省级工程技术研究中心2家。汽车零部件产业基地成为国家火炬特色产业基地。

　　（三）县域经济发展迈出新步伐

　　工业园区新增基础设施建设投资23亿元。新上固投3000万元以上工业项目70个，其中亿元以上26个。黑山生物科技和农机装备制造、北镇工业炉及配套和食品加工、凌海农产品加工、义县精细化工和冶金等产业集群累计实现销售收入1000亿元。

　　农业生产能力增强。粮食产量50.7亿斤。蔬菜、水果、肉蛋奶、水产品产量分别达到356.7万吨、935.1万吨、149.6万吨和42.5万吨，位居全省前列。新增设施农业8万亩、节水滴灌19.8万亩，治理水土流失32.4万亩。新建国家级粮油高产示范区58个。农机耕种收和水稻生产全程机械化综合水平分别达71%和90%。农业科技推广体系、农产品检验检测体系覆盖全市。畜产品质量安全事故防控、动物卫生监管信息化工作取得成效。气象预报预警和防灾减灾能力进一步提升。

　　农业产业化经营步伐加快。凌海市被农业部认定为国家农业产业化示范基地。新增龙头企业101家。12个10亿元以上重大农产品加工项目顺利推进，黑山百锦盈食品、北镇五峰米业谷物精深加工、凌海宏基园菊苣糖、义县国大乳业奶粉加工等项目即将投产。辉山乳业集团总投资70亿元的全产业链乳品产业集群项目、总投资10亿元的良种奶牛繁育集群项目分别落户黑山、义县。“一县一业”建设取得新进展。

　　城乡建设用地增减挂钩、未利用地开发、海陆统筹用地取得明显成效，保证了发展用地需求。

　　县域城镇化进程不断加快。房地产开发面积达到358万平方米，增长35%。

　　（四）服务业和城区经济蓬勃发展

　　向城区下放经济社会管理权限67项，城区发展活力增强。

　　现代服务业集聚区建设步伐加快。中央十里商街三大核心商圈基本形成，亿隆国际、温州名购、五金机电城、家乐汇等项目即将投入运营，太阳广场、红星美凯龙、银河广场、大连港总部大厦等项目开工建设。关外古玩第一城、茂业百货、蓝天百脑汇等9个项目列入省服务业100个重大项目计划。锦州银行、恒大物流等4户企业被省授予服务业领军企业。

　　城乡消费市场活跃。夜经济、假日经济持续升温。北方农展会、古玩节、汽车展、房交会等品牌展会效益创历史新高。“万村千乡市场工程”覆盖面进一步扩大。旅游规划深入实施，温泉旅游、乡村旅游、沟域旅游初具规模。全市接待旅游人数3315万人次，增长30%；旅游总收入308亿元，增长31.5%。房地产开发投资额、商品房销售额分别增长36.1%、19.4%。锦州机场开通2条国际和地区临时航线，旅客吞吐量达19万人次。

　　积极推进区域金融中心建设。五峰米业等4家企业上市工作进展顺利。义兴铁塔、天鹅焊材在辽宁股权交易中心首批挂牌。光大银行和招商银行锦州分行挂牌营业，全市银行金融机构达到11家。锦州银行在鞍山、朝阳设立分支机构。47家小额贷款公司获准开业。全年新增贷款120亿元。

　　（五）改革开放取得新成果

　　规划重组国有优势企业，盘活存量资产，华信集团、城投公司资产规模不断壮大，监管水平和融资能力进一步增强。创新融资模式，综合利用短期融资、中期票据、私募债、城投债、县域集合债等融资方式，非信贷融资实现重大突破。新组建了锦州粮食产业集团和航空物流集团。工商和质监管理体制调整、食品药品监管体制改革稳步实施。

　　加大对外开放力度，与新兴际华、韩国SK等一批国内外知名企业建立全面合作关系。万达广场、理文纸业和化工、恒力纺织工业园、国测石墨科技产业园、义县再生资源产业园等一批重大项目顺利签约。

　　成功承办了第十二届辽宁台湾周，被省政府确定为辽宁台湾周永久举办城市。锦州外经贸产业投资说明会签约项目27个，协议投资额22.8亿美元。与省外经贸厅签署了《国家加工贸易梯度转移重点承接地合作共建框架协议》。石化、光伏产品出口基地晋升为省级出口产业基地。

　　（六）城市规划、基础设施和生态环境建设不断加强

　　坚持规划先行。城市总体规划修编工作全面启动。沿海经济带、龙栖湾海洋城、城市综合交通体系、十里商街、城市水系等10项重点规划编制完成，初步形成了覆盖锦州全域的规划体系。城市空间布局不断优化，空间价值进一步提升。

　　锦凌水库下闸蓄水，几代人的梦想变为现实。60米高程以下移民搬迁工作全部完成，14个新建移民安置点具备入住条件。

　　城市基础设施建设成果显著。广州街公铁立交桥、市府西路、解放东路、城市滨河路、中央南街立交桥东西主线，以及阜盘高速锦州段60公里续建工程竣工通车。滨海大道凌海东联段工程进度过半。大中修改造公路639公里，国道102线北镇东环路续建工程完工。锦凌水库净水厂开工建设。

　　市容市貌明显改观。维修道路226条，增设维修交通标志牌2376块，增设垃圾箱1500个。摆放花箱1930个，栽植各类树木82.8万株。4个城市出入口绿化、美化全部完成。城市建成区绿化覆盖率达到40%，比上年提升5个百分点。城市医疗垃圾全部集中处置。街路网格化管理逐步完善，城区环卫保洁不断加强。

　　青山工程取得实效。清退“小开荒”17.4万亩，超坡地还林4.1万亩，围栏封育里程1109公里，人工造林47.8万亩。

　　碧水工程顺利推进。小凌河水生态环境治理城区段一期工程开工建设。西大桥段生态休闲健身广场、凌河区段堤防优化工程基本完成。大凌河治理义县城区段一期和凌海城区段工程全部完工。凌河退耕封育18万亩。城市第二、第三和龙栖湾污水处理厂建成投入运行，百股河截污干管基本建成。主城区生活污水处理能力达到95%以上。

　　蓝天工程全面实施。新购置清洁能源公交车151辆。节能热电有限公司热源厂、汤河子工业园区气化工程前期工作全面启动。城市空气质量达标率达到95%。

　　农村环境综合整治工作全面展开。完成畜禽养殖污染治理项目10个，新建垃圾处理场点182个、污水处理设施10个，新增“一池三改”用户1万户，新建绿化新村120个，硬化村内道路1075公里。

　　（七）民生工程和社会事业全面推进

　　城镇实名制就业8.2万人，城镇登记失业率控制在2.5%以内，零就业家庭继续保持动态为零。省定“五大保险”指标超额完成，医疗保险基本实现省内定点医院异地就医“一卡通”。

　　新建养老服务中心2个，新增养老床位850张。60-69岁老年人半价乘坐公交车。残疾人基本生活保障、康复服务等10项救助工程收效明显。建设保障性安居工程住房7860套（户）。完成200万平方米老旧小区综合整治。新增社区办公用房1.5万平方米。完成21个乡改镇、镇改街道工作。14.6万农村低收入群众年人均收入2500元以上。解决了9.45万农村人口饮水安全问题。

　　新建改建乡镇中心幼儿园、村办园35所。职教园区主体工程竣工。凌河区通过省基础教育强县（市）区工作验收。新农合保障水平显著提高，基本药物制度覆盖面进一步扩大。居民健康卡、健康云服务项目顺利推进。免费孕前优生健康检查年度任务圆满完成，计划生育特殊困难家庭救助收效明显。

　　世博园被授予“辽宁省文化产业示范园区”。奉国寺申报世界文化遗产工作全面启动。城区有线电视数字化整体转换工作基本完成。成功承办了十二运羽毛球、拳击赛事。

　　城区巡防体系建设进一步加强，社会治安持续稳定。圆满完成了以十二运、世园会安保为重点的维稳工作。公安机关服务软环境建设十三项措施赢得良好社会反响。律师品牌所、社区矫正、法律援助等工作成果显著。安全生产、食品药品安全、价格监管、群众信访等工作进一步加强，平安和谐锦州建设迈出新的步伐。

　　（八）政府行政效能不断提高

　　自觉接受人大法律监督、政协民主监督和社会各界监督，坚持定期向人大和政协报告、通报工作，积极采纳各民主党派、工商联、无党派人士意见。全年共办理市人大代表建议和政协提案386件，见面率、办复率均达到100%。

　　法治政府建设取得新进展，行政执法行为进一步规范。全面推进政府投资跟踪审计和经济责任审计，落实政府债务审计、财政预算执行审计整改措施，审计监督职能不断强化。严格机构编制管理，财政供养人员编制零增长。新建乡镇、街道公共服务中心106个，取消调整行政审批事项46项，行政审批效能明显提升。

　　坚持深入基层、深入群众解决实际问题，政府系统作风建设进一步加强。开展廉政和效能监察，公车私用、公款宴请、铺张浪费现象得到有效遏制。公共资源交易等“五大系统”建设全部完成。

　　国防动员、边海防建设、人防和民兵预备役工作取得新进步，双拥模范城创建深入实施。民族宗教、国家安全、妇女儿童、外事侨务、慈善救助、应急管理、防震减灾、地方志、住房公积金等工作不断加强。

　　各位代表，过去的一年，我们经受了多方面的挑战，实现了经济社会平稳健康发展。这些成绩的取得，是省委、省政府和市委正确领导的结果，是市人大、市政协和社会各界有效监督、有力支持的结果，凝聚着锦州310万人民的心血和汗水。成绩来之不易，需要倍加珍惜。在此，我代表市政府，向在各个领域和岗位上辛勤劳动、做出贡献的全市人民表示崇高的敬意！向给予政府工作大力支持的人大代表、政协委员、各民主党派、工商联和各人民团体，向关心和支持政府工作的离退休老同志，向参与锦州发展建设的中省直各部门、各单位，向驻锦部队和武警官兵，向所有关心支持锦州发展建设的海内外朋友，表示衷心的感谢！

　　回顾过去一年的工作，我们也清醒地认识到，发展中还有不少差距和问题。一是财政收入结构不合理，涉土税收、非税收入占比较大，主体税种收入贡献不足；二是服务业占比只有36.1%，调结构的任务还很艰巨；三是外贸依存度只有15.3%，低于全省近12个百分点；四是工业产业集群发展迟缓，缺少具有牵动作用的重大项目，还没有形成对全市经济的支撑作用；五是城镇化率只有51.8%，低于全省近14个百分点；六是区域间发展不平衡，沿海突破战略作用还不明显。同时，社会管理与公共服务水平亟待提高，改善民生的任务仍然繁重，政府作风建设还有待加强。对于这些问题，我们要高度重视，认真采取措施，切实加以解决。

　　二、2014年工作安排

　　今年是全面深化改革的第一年。我们要牢牢把握这一历史机遇，进一步释放改革红利，团结一心、扎实工作，继续保持经济稳中有进的良好态势，不断开创改革发展新局面。

　　今年政府工作的总体要求是：全面贯彻落实中央和省经济工作会议、市委十一届六次全会精神，坚持稳中求进、改革创新，着力激发活力，转方式调结构，保障改善民生，提高经济发展质量和效益，为加快推进辽宁沿海第二大城市建设奠定更加坚实的基础。

　　全市经济社会发展的主要预期目标是：地区生产总值增长9%，固定资产投资增长18%，公共财政预算收入增长9.5%，规模以上工业增加值增长11%，社会消费品零售总额增长14%，实际利用外资增长10%，引进省外资金增长10%，出口创汇增长10%，城镇居民人均可支配收入增长9%，农民人均纯收入增长12%，居民消费价格总水平涨幅控制在全省平均水平以下，全年95%以上天数空气质量达到国家二级标准。

　　（一）全面深化各项改革

　　根据省里部署，今年要统筹推进七个方面改革工作：

　　推进国有企业改革。进一步完善出资人制度、规范国资国企监管运营，加快国有资产规划重组，组建文化、旅游、交通、供热、公用基础设施建设集团，加大公司化改造力度，全面提高产业集中度和企业竞争力，促进国有资产保值增值。妥善解决好改制企业遗留问题。

　　推进政府职能转变和机构改革。大力简政放权，最大限度减少行政审批事项，减少预审和前置审批环节。以加强事中事后监管为重点，改进政府管理。强化政府机构和职责整合，启动市、县政府机构改革。积极稳妥推进事业单位分类改革。改进政府绩效考评工作机制和指标体系。加快推进10个经济技术开发区、产业园区管理体制和运行机制创新。

　　推进财税体制改革和价格改革。加快公共财政体系建设，完善预算管理制度。完善税收征管体制，建立健全政府非税收入收缴分离改革管理制度。注重税源培养，加强税收征管，切实改变财政收入增速缓慢、结构不合理、质量不高的问题。做大做强华信、城投等国有资产投资公司，增强投融资能力，突破资金瓶颈。建立财政统筹的归口管理机制和融投建管还的风险管控机制，有效防控政府债务风险。全面深化水、电、燃气价格改革。

　　推进农村土地制度改革。积极探索农村集体经营性建设用地进入市场、节约集约用地专项奖励基金和土地出让履约保证金等改革试点工作。大力推进青山、碧水、蓝天工程，建立生态补偿机制和排污权交易平台。加快推进户籍制度改革，加快太和区、开发区和松山新区全域城镇化建设步伐。

　　推进市场体系建设改革。全面做好工商登记制度改革。进一步整顿和规范市场中介机构，建立公平竞争的市场秩序，激发市场主体活力。培育壮大各类生产资料市场和生活资料市场，加大金融、人才、技术等生产要素市场建设力度。

　　推进教育、科技、卫生和文化体制改革。统筹城乡义务教育资源均衡配置，加快现代职业教育体系建设。支持企业自主创新，加快建立产学研协同创新机制。加快基层医疗卫生机构综合改革步伐，稳步推进公立医院改革。完成市直国有文艺院团改革，支持社会资本参与文化产业发展，加快打造国家级古玩交易中心，建设包装印刷产业基地。

　　推进城市管理体制改革。全面下放城市管理权限，进一步明确城区政府属地化管理的主体责任，尽快建立起“两级政府、三级管理、四级网络、条块结合、以块为主”的新型城市管理体制，建立“纵向到底、横向到边、分工明确、责任到人、奖惩分明”的网格化城市管理长效机制。

　　（二）强力推进重大基础设施建设

　　完成锦凌水库附属工程建设，推进水土保持及环境保护工程，尽快发挥生态和经济、社会效益。完成锦凌水库净水厂主体工程。

　　加快锦州湾机场建设，确保年底具备通航条件。开工建设机场一、二号主干路和小凌河东支流大桥工程。统筹推进水电暖气等配套设施建设。启动航空物流城建设。

　　确保滨海大道凌海东联段工程建成通车。启动龙栖湾疏港铁路、公路和大凌河入海口右岸滨河路建设。

　　加快锦州港西部海域防波堤、煤炭码头工程建设，实现货物吞吐量9000万吨、集装箱运输100万标箱。完成龙栖湾港区一港池2个通用泊位、3万吨级航道等工程建设，实现简易通航。

　　开工建设华润锦州电厂2台66万千瓦机组项目。

　　实施城市畅通工程，开工建设中环西路、南广西路、滨河路二期、重庆东路、士英街铁路桥拓宽改造、广州街跨小凌河大桥等工程，打通林西街、吉祥街、徐州街等断头路，打造锦娘线城市景观路。

　　启动京沈高速公路与黑山县连接路、国道102线沟帮子外环路工程。完成义县外环路续建工程。

　　完成省下达的京沈客专锦州段拆迁征地任务。

　　（三）全面提升沿海经济带发展水平

　　加快推进沿海基础设施建设。完善重点工业园区道路管网等配套设施，提升公共服务功能。整体搬迁改造开发区5个村屯。辽宁医学院、锦州师专、锦州中学等迁建工程全面开工。

　　加快重大项目引进建设。着力推进开发区化工产业园、龙栖湾化纤纺织工业园、松山健康产业园、汤河子中国“钛都”等重点园区建设，力促理文纸业和化工、恒力纺织工业园等重大项目开工建设，确保中北石化60万吨润滑油、宏利达糖业6万吨食品、仁禾生姜加工等项目建成投产。

　　加快滨海现代服务业发展。创新管理体制和运营机制，确保世博园良性运营。加快白沙湾温泉休闲度假区、大有温泉生态城等项目建设，积极争创国家级旅游度假区。争取世界温泉与气候养生协会永久论坛和中国海洋经济博览会项目落户锦州。确保王府井·滨海新天地、亿达希尔顿酒店、东北亚国际物流基地等项目开工建设。

　　（四）全力推动工业经济稳定增长

　　深入实施工业五项工程。计划安排工业固定资产投资亿元以上项目100个，其中10亿元以上33个；5000万元以上竣工投产项目100个；新兴产业项目100个。完成工业固定资产投资520亿元。

　　全力推进工业“6921”工程。围绕以石化及精细化工等为重点的6大产业集群，以开发区西海工业园区等为重点的9个工业园区，加快培育壮大锦州石化分公司等21个龙头企业。集中政策倾斜和要素保障，确保一批重点项目尽快开工投产，尽快形成对全市的支撑能力。5个省重点产业集群实现销售收入1520亿元，增长24%。大力发展工业地产，确保工业地产取得突破。

　　强力推进光伏产业发展。确保瑞德兴阳100MW太阳能芯片、阿特斯400MW太阳能电池组件、东旭集团200MWp地面光伏电站等项目开工建设。加快奥克阳光、赛瑞新能源等5个项目二期工程建设。完成国家住建部20MWp分布式光伏电站项目建设。

　　抓好工业经济运行。加强对重大项目、重点企业的跟踪服务，提供要素保障。鼓励各类投资主体参与工业园区基础设施建设。搭建银企对接平台，组织好地工产品产销衔接。设立工业发展专项资金。

　　加快高新技术企业和创新型企业培育。积极推进产学研合作，建立完善各类研发服务平台。着力推进国家硅材料及光伏、健康产业、汽车零部件特色产业基地建设。支持辽宁工业大学科技园申报国家级科技园，争取高新区升级为国家级高新区。

　　（五）加快县域经济和现代农业发展

　　县域经济要为全市经济发展做贡献，确保经济总量、公共财政预算收入在全省位次前移。

　　加强工业园区建设。围绕主导产业集群，着力抓好项目引进、开工和投产。确保新上3000万元以上工业项目100个，每个县（市）形成1-2个销售收入超百亿元的工业产业集群。

　　推进现代农业发展。积极创建国家现代农业示范区，大力培育现代农业经营主体。新增设施农业10万亩、粮油高产示范区60个、农业产业化龙头企业100家、农民专业合作社200个、家庭农场1000个。加快推进黑山蛋鸡、北镇葡萄、凌海外贸菜、义县奶牛产业等“一县一业”建设。力争每个县（市）新上1-2个10亿元以上重大农产品加工项目。

　　改善农村生产生活条件。完成节水滴灌16万亩，治理水土流失32万亩，改洼治涝15万亩。耕种收综合机械化水平达到73%以上。推进畜禽标准化养殖场建设，强化动物防疫和畜产品安全监管。推进气象突发事件预警信息发布系统建设，不断提升气象公共服务水平。

　　强化耕地保护，集约节约用地。用好新增建设用地指标，做好存量土地挖潜、城乡建设用地增减挂钩等工作。

　　加快县域城中村、城边村、老旧小区拆迁改造，推进新城、新市镇建设，完成县域房地产开发面积400万平方米。

　　（六）大力发展城区经济和服务业

　　大力发展服务业是调结构的方向，也是稳增长、惠民生的重点。要科学规划，突出重点，加强协调，大力营造支持服务业发展的浓厚氛围。到“十二五”末，服务业占比要达到全省平均水平。

　　推进重点服务业项目建设。启动中央十里商街拆迁改造，为重大服务业项目落地创造条件。加快推进太阳广场、深圳茂业、银河广场、红星美凯龙、蓝天百脑汇、大连港总部大厦项目建设，力争万达广场项目上半年开工。

　　培育新的服务业增长点。大力发展物流、电子商务等10大重点产业。继续办好汽车展、房交会等品牌展会。加快县域服务业集聚区建设。做强做大北镇窟窿台蔬菜、黑山杂粮加工等大型专业批发市场。继续推进“万村千乡市场工程”、“农超对接工程”和乡镇商贸中心建设。加快推进滨海旅游、温泉旅游、沟域旅游和乡村旅游发展。采取有效措施，保持房地产市场健康发展。

　　加快区域金融中心建设。争取有2家域外银行落户、3户企业上市。实现新增贷款130亿元。鼓励发展小额贷款公司，加大对中小微企业贷款扶持力度。

　　以第三次全国经济普查为契机，建立健全服务业统计体系。

　　（七）全面扩大对外开放

　　充分利用国家加工贸易梯度转移重点承接地政策优势，坚定不移地实施对外开放战略，扩大开放领域，优化开放结构，提高开放质量，用高水平的开放促改革、促发展、促转型。

　　坚持内商外商齐招，内资外资并引，精心组织好面向长三角、珠三角区域，以及港台、日韩、欧美等国家和地区的招商推介活动，力争在主题概念、主导产业招商和引进研发团队上取得新突破。大力发展外向型经济，做强做大1个国家级、5个省级开发区。规划建设台商大厦、台湾产业园、台湾商品一条街，全面提升辽宁台湾周永久举办地的水平和影响力。

　　大力开拓国际市场，着力提高外贸依存度，加快建设外贸出口大市。加强与蒙古国经贸合作，推进锦州港出海口建设。全面优化对外开放软硬环境，加快推进海关特殊监管区域建设，积极争取在锦州设立自由贸易区，提高服务开放的能力，提升开放型经济发展水平。

　　（八）统筹推进城乡生态环境建设

　　建立青山长效保护机制。全面推进3年大规模造林绿化工作，完成沿海、城北和环西北边界“三带”防护林25万亩，经济林产业带25万亩，南山、锦凌水库周边新植苗木170万株，以及“千村”绿化和渤海大道、龙栖湾大道、嵩山路绿化工程。

　　全面开工建设城市水生态环境综合治理工程。规划建设大凌河生态文明示范区。坚持开发与保护并重，切实加强海洋环境监管与保护。结合棚户区改造，有计划“退楼还绿、拆墙透绿”，增加住宅区街心花园、健身广场。确保3座新建污水处理厂稳定运行，启动中水回用管线铺设工程。

　　深入实施蓝天工程。开工建设2座热源厂，逐步拆除分散燃煤锅炉。加快推进燃气管网和分输站建设。完成现有加油站、储油库油气回收改造。淘汰黄标车，推广新能源汽车。新建城市建筑垃圾填埋场1座。

　　（九）着力保障和改善民生

　　重点办好10件民生实事。主要包括：

　　发展义务教育。完成100所义务教育学校标准化和教育信息化“三通两平台”建设。

　　提供医疗保障。提高新农合筹资标准，达到年人均400元；在全市1147个村卫生室、20个社区卫生服务中心、115个社区卫生服务站部署健康管理云终端，形成医疗健康信息一体化服务网络；实现计划生育免费孕前优生健康检查和免费技术服务目标人群全覆盖。

　　发展文化事业。开展“美丽锦州、幸福歌潮”群众性主题文化广场演出100场，“送戏下乡”惠民演出200场；建成城市社区书屋79个。

　　推动全民健身。新建农民健身工程20个、城市社区健身路径 100套。

　　加强就业工作。实现实名制就业4.1万人，城镇登记失业率控制在3%以内。

　　强化社会保障。城乡低保、农村五保对象救助标准分别提高10%以上；残疾人医疗、养老保险参保率达到95%以上；确保6万农村低收入群众年人均收入达到3200元以上。

　　大力推进棚改。安排建设保障性安居工程住房14150套（户），搬迁改造21个城市棚户区11588户。

　　建设公益设施。充分利用现有资源，建设辽西大剧院、市博物馆新馆、青少年宫、城市规划展示馆等公益性建筑；开工建设市养老综合服务中心和第三军休服务管理中心；新增社区办公用房1.4万平方米，社区办公用房全部实现200平方米以上；新建城乡公益性公墓20处。

　　改善城市环境。新建配水池遗址、768、新制北里、士英街等4处绿地公园；完成200万平方米老旧小区综合整治任务。

　　治理农村环境。全面完成村庄亮化工程；完成2座农村客运站、40个农村客运候车亭建设，大中修县级公路379公里；解决6.37万农村人口饮水安全问题。

　　（十）切实加强政府自身建设

　　自觉接受人大法律监督、工作监督和政协民主监督，坚持重大事项向人大报告和向政协通报制度，坚决执行人大及其常委会的各项决议、决定，积极支持人民政协履行职能，认真办理人大议案、代表建议和政协建议案、提案，不断提高政府科学决策水平。

　　加强和创新社会管理。推进公安信息化应用创新，夯实公安基层基础建设，不断提升维护社会稳定、公共安全和服务经济发展的能力。加强司法行政基层基础建设，提升社区矫正、法律援助和法律服务水平。完善信访工作长效机制，切实维护群众合法权益。开展食品药品安全专项行动。全力抓好重点行业、重点领域、重点产品安全监管和隐患治理，保证人民群众的生命财产安全。

　　推进法治政府建设。深化重点领域政务公开、重大决策政务督察，不断提高行政效率和服务水平。围绕人民群众反映强烈的热点焦点问题，抓好执法监察、廉政监察和效能监察。强化政府重大投资、经济责任跟踪审计，确保资金使用效益。

　　深入开展党的群众路线教育实践活动，切实加强政府自身建设，转变职能、转变作风，勇于负责、敢于担当，不断提高领导科学发展的能力。全力营造风清气正、干事创业的浓厚氛围，形成推动改革发展的强大合力。

　　各位代表，全面深化改革的号角已经吹响，让我们在市委的坚强领导下，紧密团结和依靠全市人民，锐意进取，攻坚克难，共同谱写改革发展的崭新篇章！
